# Wildlife, Reservoir of Zoonotic Agents: Moving beyond Denial and Fear

**DOI:** 10.3390/pathogens12091081

**Published:** 2023-08-25

**Authors:** Bernard Davoust, Younes Laidoudi

**Affiliations:** Veterinary Research Center of the Mediterranean Infection University Hospital Institute, Aix-Marseille University, 13005 Marseille, France

Human infections that originate in animals are quite frequent and warrant further investigation. According to the World Organisation for Animal Health (WOAH), 60% of human infections are zoonotic, and 75% of emerging human infectious diseases are zoonoses. The recent coronavirus pandemic forcefully drives home this fact. Some zoonoses, such as salmonellosis, leptospirosis, and rabies, are frequent and widespread in most countries. Others, like arboviruses and glanders or the plague, are rarer or more geographically localized. Zoonoses vary significantly in terms of their medical severity. Some are occasionally fatal, such as viral encephalitis and Ebola. Others, like Lyme disease, are subjects of scientific media controversy. Antibiotic-resistance can, in some respects, be considered as a source of zoonosis and treated as such. The WOAH also notes that 72% of the 60 emerging infectious diseases that have an animal origin or reservoir have an origin in wildlife. In addition, the 144 human diseases originating in wild animals have become significant for human health over the past 60 years.

Ever since wild animals (primarily bats) were suspected of being the cause of the COVID-19 pandemic, the general public has become familiar with the notion of zoonosis. However, infectious diseases transmissible from animals to humans (and vice versa) have long been the subject of in-depth studies. Rabies remains the most emblematic, given its lethality. Today, the holistic approach to global health is partly based on the One Health concept, which is being increasingly integrated into public policy. This concept addresses the interactions between humans, animals, plants, and the environment as health issues.

Regarding zoonoses, wildlife serves as a reservoir of pathogens for both domestic animals and humans, and this requires careful assessment. Furthermore, animals can act as sentinels for the circulation of infectious agents within each ecosystem. Therefore, we urgently need to advance and disseminate knowledge concerning wildlife as a reservoir of zoonotic agents. Multidisciplinary teams on every continent are currently conducting studies on this topic.

This Special Issue of the journal *Pathogens,* entitled “Surveillance of Zoonotic Pathogens Carried by Wildlife”, comprises 17 articles contributed by authors from 16 countries across Europe, Asia, and America. Among these articles, twelve studies focus on mammals, while the remaining five pertain to birds.

One of the comprehensive reviews, authored by Jain et al., presents the history of Ebolavirus epidemics in Africa since 1976. This review delves into topics such as etiology, natural hosts, zoonotic reservoirs, and transmission mechanisms [1]. Another review addresses zoonotic diseases of hedgehogs. Jota Baptista et al. conducted a meta-analysis of twelve studies on this subject, encompassing both urban and rural areas of Europe [2].

The other fifteen articles are original contributions. Four studies are dedicated to arboviruses. In Romania, Coroian et al. employed serology to demonstrate the circulation of the West Nile (WNV) and Usutu virus (USUV) in migratory birds [3]. Similarly, in Spain, Casades-Marti et al. detected the WNV in wild birds using serology and PCR, correlating the results with various factors like bird diversity, vector presence, and climate [4]. Mancuso et al. investigated ticks collected from migratory birds on three Italian islands. Their tests revealed the presence of Crimean–Congo hemorrhagic fever virus (CCHFV) and WNV in the African tick *Hyalomma rufipes* [5]. In India, Singh Malik et al. reported the detection and characterization of the rotavirus C genome in sloth bears (*Melursus ursinus*) residing in urban areas, possibly originating from pigs or humans [6].

Additionally, Reinhardt et al. studied a hundred raccoons (*Procyon lotor*) in Germany, screening blood and organs via qPCR. Some samples tested positive for carnivore protoparvovirus 1, canine distemper virus, *Leptospira* spp., and *Anaplasma phagocytophilum*. However, they tested negative for WNV and the influenza A virus [7]. We conducted a seroepidemiological survey for Aujeszky’s disease virus in 399 wild boars from southeastern France, revealing a high prevalence of 30% [8].

Leptospirosis, a significant bacterial zoonosis, is addressed in the context of hedgehogs and raccoons [2,7]. It also forms the focal point of three other field studies. Haring et al. collected samples from three shrew species (N = 372) and hedgehogs, primarily within Germany. Their findings encompassed the detection of *Leptospira kirschneri*, *L. interrogans*, *Anaplasma phagocytophilum*, *Neoehrlichia mikurensis*, and two *Bartonella* strains [9]. Harran et al. identified *Leptospira kirschneri* of the pathogenic serogroup Grippotyphosa in water voles (*Arvicola terrestris*) inhabiting ruminant pastures in central France. With a serological prevalence of 76%, the exact epidemiological role of voles in sheep leptospirosis within this ecosystem remains uncertain [10]. In Malaysia, Shafie et al. discovered PCR evidence indicating that 20% of the rodents in recreational areas carried pathogenic *Leptospira* [11].

In Italy, Carrera et al. studied the spatiotemporal distribution of *Salmonella enterica* in 212 naturally deceased hedgehogs, revealing an estimated overall prevalence of potentially zoonotic *Salmonella* of 39% [12]. In the USA, wild boars (*Sus scrofa*) undergo specialized epidemiological surveillance for *Brucella suis* swine brucellosis. Brown et al. conducted experimental infections to enhance serological diagnosis through the buffered acidified plate antigen test or the *Brucella abortus/suis* complement fixation [13]. Meanwhile, in Germany, Burgold-Voigt et al. isolated a *Staphylococcus aureus* strain from a badger (*Meles meles*) and characterized its genome, noting its lack of antibiotic resistance genes. Utilizing three bacteriophages, the strain exhibited virulence and successfully infected other hosts [14].

Enterococci serve as excellent indicators of antimicrobial resistance. Kwit et al. assessed the prevalence of *Enterococcus* spp. in wild birds in Poland, revealing that 10% of *E. faecalis* cases and 50% of *E. faecium* cases displayed resistance to antimicrobial agents upon susceptibility testing [15]. In southeastern France, we demonstrated a colistin resistance mechanism within *Enterobacter hormaechei* subsp. *steigerwaltii* isolated from wild boars (*Sus scrofa*) [16]. Lastly, Dini et al. identified *Toxoplasma gondii* infestations in wild migratory birds passing through Italy, potentially acting as intermediary hosts for the spatial dissemination of toxoplasmosis. The overall positivity rate was 14% (7/50) [17].

This endeavor, involving the collection and analysis of field data, enables us to have a deeper understanding of specific health risks. This sharing of knowledge invites us to delve deeper into international politics, media coverage, and public opinion concerning wildlife. Our intention is to embrace the intricate reality that threads through all the articles in this Special Issue. Indeed, wildlife is vulnerable and intricately interdependent, particularly within the realm of human activity. As public authorities assume the responsibility for safeguarding these creatures, they must also engage in a comprehensive exploration of their ecological aspects as well as their roles as reservoirs for known or emerging zoonotic agents.

Remarkably, this health-oriented approach is not sufficiently addressed by the International Union for Conservation of Nature (IUCN). Although these animals are sanctuarized, the IUCN seems to adopt an ideological stance of denial. This became evident during the World Conservation Congress held in Marseille (France) in 2021. Infectious diseases within wildlife were scarcely addressed, with only one declaration occurring in a statement issued by the Wildlife Health Specialist Group of the Species Survival Commission within the IUCN, which comprises scientists including veterinarians [18]. Nevertheless, we believe their statement is biased, as it essentially makes human beings the sources of infection for animals. Regrettably, it downplays the two-way concept, which emphasizes wildlife as potential sources of zoonoses, while sidelining domestic animals [19].

It is particularly disappointing that the IUCN’s statement does not explicitly advocate for the monitoring of wildlife as infection reservoirs by studying the circulation of zoonotic agents within biodiversity [20]. Thus, we emphasize the crucial role of veterinarians in advancing our knowledge by responsibly collecting biological samples from wildlife across various habitats. These scientists adeptly utilize proportionate access methods such as capture and release, along with non-invasive sampling (e.g., feces), while prioritizing animal welfare. Rather than evading ideological considerations, we could get to the roots of zoonoses and even of pandemics. In essence, the media’s globalized portrayal of mankind’s culpability hampers the progress of science and public health. In stark contrast to denial, public sentiment occasionally veers towards fear and the unjust stigmatization of wildlife. During the COVID-19 crisis, we even heard extremist voices advocating the extermination of bats and pangolins. Fortunately, such misguided notions have had minimal impact in the field and have not harmed biodiversity.

Amidst the evolving relationship with wildlife, poised between denial and fear, it is imperative for scientists to immerse themselves further in zoonosis research. They recognize the inherent risks they undertake by closely investigating the circulation of infectious agents in both wild and domestic animals. The enchanting appeal of nature underscores the need to safeguard it, while also treating the conservation of species in a way that does not compromise human and/or animal health.

We hold the belief that the WOAH champions a pragmatic approach, considering wild animal species through the lens of thoughtful protection within their habitats, while also acknowledging their role as reservoirs (or sentinels) of infectious agents that need to be monitored [21]. The WOAH underscores the requisites for infectious disease management: regulatory frameworks, trade embargoes, careful control, population management, global alignment of health regulations and diagnostic tools. Free from idealistic notions of biodiversity, governments and international scientific organizations must sustain their efforts to facilitate interactions and foster productive dialogues among zoologists, ecologists, veterinarians, and medical professionals, characterized by ever-improving interpersonal dynamics.

The alliance between humanity and its environment finds enthusiastic support in the One Health approach championed by the authors of the articles in this Special Issue. Their collective endeavor enhances our understanding of shared and distinctive facets of each situation they present, offering diverse perspectives on the surveillance of zoonotic pathogens carried by wildlife. Our gratitude extends to them for their confidence, as well as to MDPI, the publisher of *Pathogens*. With their array of viewpoints, they illuminate our comprehension, enabling us to unbiasedly survey the zoonotic pathogens carried by wildlife.
